# Cross-sectional analysis of adverse outcomes in 1,029 pregnancies of Afro-Caribbean women in Trinidad with and without systemic lupus erythematosus

**DOI:** 10.1186/ar2332

**Published:** 2007-11-27

**Authors:** Mariam Molokhia, Noreen Maconochie, Alan Leslie Patrick, Pat Doyle

**Affiliations:** 1 Non-Communicable Disease Epidemiology Unit, London School of Hygiene & Tropical Medicine, Keppel Street, London WC1E 7HT, UK; 2 Kavanagh Street Clinic, Port of Spain, Trinidad

## Abstract

The objective of the study was to examine pregnancy outcomes in women with systemic lupus erythematosus (SLE) and population controls in Trinidad. We performed a cross-sectional analysis of adverse outcomes in pregnancies of Afro-Caribbean women with SLE and without SLE. One hundred and twenty-two female adult cases of SLE and 203 neighbourhood age-matched women without SLE were interviewed concerning details of their reproductive history, and the anticardiolipin antibody (ACL) status was established for women with SLE. A total of 1,029 pregnancies were reported (356 by women with SLE, 673 by women without SLE). In women with ≥ 1 pregnancy the total number of pregnancies was similar in women with a diagnosis of SLE and women without; however, a lower proportion of women with SLE had ever been pregnant compared with women without SLE (80% versus 91%, *P *= 0.002). In multivariate logistic regression analyses adjusted for maternal age, district of residence, pregnancy order and smoking, SLE pregnancies were more than twice as likely to end in foetal death than non-SLE pregnancies (odds ratio (OR), 2.4; 95% confidence interval (CI), 1.2–4.7). This effect was driven by a large increase in the odds of stillbirth (OR, 8.5; 95% CI, 2.5–28.8). The odds of early miscarriage (OR, 1.4; 95% CI, 0.6–3.1) and of mid-trimester miscarriage (OR, 1.9; 95% CI, 0.4–9.5) were higher, but were not statistically significantly different, in SLE pregnancies than in non-SLE pregnancies. The odds of ectopic pregnancy (OR, 7.5; 95% CI, 0.9–62.5) and of preterm birth (OR, 3.4; 95% CI, 1.2–10.0) were higher in SLE pregnancies conceived after diagnosis than in non-SLE pregnancies. There was no evidence of raised levels of IgG or IgM ACL among the majority (93/97 women, 96%) of SLE cases who reported sporadic mid-trimester miscarriage or stillbirth, although there was evidence of high levels of IgM and IgG ACL among women reporting three or more miscarriages and three consecutive miscarriages, and of raised IgG ACL among those experiencing ectopic pregnancy. In conclusion, we found evidence for a large increase in risk of stillbirth in the pregnancies of Afro-Caribbean Trinidadian women with SLE (not accounted for by high ACL status). There was some evidence of an increased risk of preterm delivery and ectopic pregnancy in pregnancies conceived after a diagnosis of maternal SLE.

## Introduction

Systemic lupus erythematosus (SLE) is a complex autoimmune disease that predominantly affects females of child-bearing age (female:male ratio approximately 9:1), with both genetic and environmental determinants, and is particularly common in Afro-Caribbean populations. SLE has a well-recognised association with second-trimester miscarriage; studies have also examined other adverse pregnancy outcomes including prematurity and pregnancy loss [1-16]. Some of these studies, however, are based on small numbers and/or tertiary referral centres with many high-risk patients, and few studies involved a comparison group [2,8].

Risk factors that have been previously identified for adverse pregnancy outcome in women with SLE (particularly second-trimester miscarriage) include antibodies to phospholipids (lupus anticoagulant and anticardiolipin antibodies), lupus activity and renal disease [1-3,5-9,11,12,17,18]. Many studies may underestimate the rates of early miscarriage, however, as patients may not have been referred after a pregnancy was lost.

Trinidad was selected as an ideal place for study of pregnancy outcomes as large family sizes are common combined with a young age of reproduction. There are no known previous such studies in the Caribbean. There are no national data for Trinidad available on miscarriages, although data are provided on other reproductive outcomes such as live births and stillbirths [19]. At the last census in 2000, the population of Trinidad & Tobago was 1.26 million. Estimating the prevalence of SLE in Northern Trinidad is difficult as we do not have exact denominator data. Typical prevalence rates in Afro-Caribbean women in the United Kingdom, however, are around 200 per 100,000 adult women, which are six to eight times higher than in Europeans [20,21]. Preliminary work carried out by the authors suggested there may be approximately 373 cases of SLE in Northern Trinidad (see Discussion), with a population denominator of approximately 170,000 adult females [22] – thus giving a crude SLE prevalence of approximately 219 per 100,000 in females in Trinidad.

Health services in Trinidad are mainly public sector with some private hospitals and clinics. Primary healthcare services include 102 nationwide health centres. Secondary healthcare is available at eight district hospitals and three large government hospitals in Port of Spain, San Fernando and Arima. Tertiary healthcare is available in Port of Spain. One of the main facilities is the Mount Hope Medical Complex, which houses a 340-bed general purpose hospital, including a 110-bed maternity hospital. Overall there is reasonable access to healthcare, even in low-income groups.

The aim of the study was to examine adverse pregnancy outcomes (miscarriage, stillbirth, ectopic pregnancy and preterm delivery) in a large group of Trinidadian Afro-Caribbean women with SLE and without SLE.

## Patients and methods

### Study design

The study was a population-based questionnaire survey of reproductive outcomes of adult Afro-Caribbean women living in Northern Trinidad diagnosed with SLE and without SLE. These women were identified for a case – control study of SLE that was conducted from 1998 to 2002 [23]. We obtained reproductive histories in addition to information on various lifestyle factors and demographic factors, including socioeconomic status, and contraceptive pill use. No women with SLE or without SLE were pregnant at the time of interview. Serum from 122 women with SLE was sent for IgG anticardiolipin antibody (ACL) and IgM ACL assays at a standard UK laboratory. (The laboratory normal range for IgG ACL was 0.0–12.0 U/ml, and that for IgM ACL was 0.0–5.0 U/ml.)

### Ascertainment of women with systemic lupus erythematosus

Cases were adult women with SLE, aged 18 years and older, resident in northern Trinidad from 1998 to 2002 who were selected according to the standard revised American Rheumatism Association 1982 criteria [24] through multiple sources. The study was restricted to women of Afro-Caribbean ethnicity (this was because the original study design was examining SLE risk in relation to African ancestry in Afro-Caribbean populations, and other ethnic groups such as Indian or Chinese show epidemiological evidence for increased risk of SLE). In summary, surviving adult women with SLE were ascertained from five sources from both the public sector and the private sector: the lupus register at Port of Spain General Hospital (started in 1992); the rheumatology, renal, and dermatology outpatient departments from the two main hospitals that serve the defined population of Northern Trinidad (Port of Spain General Hospital and Mount Hope Hospital, Arima); a systematic search of outpatient records plus hospital laboratory records of positive tests for antinuclear antibodies at the two main hospitals to identify cases of SLE; Lupus Society meetings and through advertisements in the quarterly newsletter and television; and an immunology register compiled from records for 1992–2000 that included cases diagnosed through high (> 1:256) titres of antinuclear antibodies and antidouble-stranded DNA antibodies and those patients with positive histology from skin biopsies. Details of these registries are described elsewhere [23].

More than 90% of patients with SLE registered with the Lupus Society attended one of the two main hospitals servicing this catchment area. Few patients had been seen exclusively privately (< 5%). We established that case records are available for more than 90% of these patients and that these records include adequate information on the clinical and laboratory findings upon which the patient diagnosis was based. Both hospitals were visited to assess the quality and completeness of the records. When invited for interview, patients were also asked to bring any private case records in their possession, and these were used to validate clinical criteria and medical history.

### Ascertainment of women without systemic lupus erythematosus

For each case, randomly chosen households in the same neighbourhood were sampled by the field team to obtain two women without SLE, matched with the case patient for sex and for 20-year age group (this was adjusted as the original matching for 10-year age group led to many exclusions for controls). For each case patient the interviewer drew a map, compiled a list of 25 adjacent households and visited these households in random order using random-number tables to interview all eligible individuals in the same age – sex group as the case. Where possible, a listing of the age, sex and ethnicity of all household members was obtained and arranged in descending order of age. The interviewer then selected at random (using printed tables) one suitable individual for interview according to the numbers eligible. This was continued until eligible controls had been obtained from at least two separate households.

### Patient interviews

Where possible, all cases identified through the hospital clinics and lupus registers were telephoned and invited to participate in the study. Where telephones were not listed, case records and telephone directories were checked. We established from preliminary studies that 80% of households had a telephone. Where telephones were not available, invitations about the study together with a study information sheet with contact telephone number were sent to the participant's address or were delivered by hand. Nonrespondents were sent reminders or were revisited. All women were offered a choice to be interviewed at home or in the project office using a validated questionnaire that included demographic items, socioeconomic indices in childhood and adult life, birth order, medical history, and reproductive history. Detailed (self-reported) information was collected on all pregnancies, including date of pregnancy end, gestation and outcome of pregnancy. We did not have access to accurate birthweight records so this parameter could not be studied. Pretesting of the questionnaire was used to train research interviewers and to refine ambiguous questions. Participants were paid travel expenses, and were offered a complimentary full blood count, cholesterol measurement, or membership to the Lupus Society for 1 year. All reasons for nonresponse in relation to obtaining the household listing or in relation to selecting suitable control individuals were documented. Crude socioeconomic data on status of nonresponders were compared with those of responders where possible (type of housing, car and telephone ownership). Up to two return visits were made at different times of the day, including weekends and after working hours, in order to interview potential control individuals. Blood was taken for DNA and serum storage prior to analyses at the time of interview.

Ethical approval was granted by the Ethics Committee of the London School of Hygiene & Tropical Medicine and by the Ministry of Health of the Republic of Trinidad & Tobago. All participants provided informed consent for this study.

### Pregnancy outcome: statistical analyses

The majority of the analyses described here are pregnancy based. Women were excluded from this analysis if they were aged younger than 18 years at interview (one woman with SLE) or were too ill to participate (four women with SLE). The outcomes of interest were live birth, preterm birth, miscarriage (early and late), ectopic pregnancy, stillbirth, and foetal death as a whole (comprising all miscarriage and stillbirth). The exposure of interest was maternal SLE status. Three categories of foetal death were used according to the reported gestation at the end of the pregnancy: early (first-trimester) miscarriage (≤ 12 completed weeks), late (mid-trimester) miscarriage (13–23 weeks), and stillbirth (≥ 24 weeks gestation). Preterm delivery was defined as a livebirth that ended before 37 weeks of completed gestation.

The denominator for the analyses of early miscarriage was all reported pregnancies; for late miscarriage, the denominator included all pregnancies surviving more than 12 weeks; and the denominator for stillbirth included all live and stillborn babies. For analyses of ectopic pregnancy, the denominator was all pregnancies; and for analyses of preterm delivery, the denominator was all live births. Ectopic pregnancies were excluded from all analyses of foetal death. Information on termination was incomplete as termination for nonmedical reasons is illegal in Trinidad, and was therefore omitted from all analyses. Multiple pregnancies (a total of five twin pregnancies, which could only be identified for those women with gestation over 24 weeks) were excluded from all analyses.

All analyses were carried out using STATA statistical software (release 9.0, 2005; Stata Corporation, College Station, TX, USA). All analyses were adjusted for maternal age at the time of conception, for pregnancy order and for smoking. Although the original design was matched on the neighbourhood of residence, results were similar using matched or unmatched analysis – the latter results are therefore presented. The district of residence, however, was included in all models since in the original design women without SLE were matched to SLE cases in the same neighbourhood as the SLE cases. Analyses were performed using different markers of socioeconomic status, including years of education and district of residence. The unit of analysis was a pregnancy. The association between the relevant pregnancy outcome and SLE status at time of survey or between the relevant pregnancy outcome and SLE status at time of conception was explored using logistic regression analysis – effects on risk being estimated by odds ratios (ORs) with 95% confidence intervals (CIs). Since women could have more than one pregnancy in the analysis, a robust method based on the 'sandwich estimate' [25] was used to compute standard errors, with Wald tests used to test the statistical significance of parameters [26].

## Results

The response rate for eligible SLE cases in the case–control study was 122/131 (93%). Two women with SLE refused to participate and seven did not attend for interview. Of eligible women without SLE, the response rate was 70% (203/290). Initial comparison of nonresponders in women without SLE did not show significant differences in age or socioeconomic status. There was no association of district with risk of SLE.

### Woman-based analyses

Table 1 presents data for the characteristics of the 122 female participants with SLE and of the 203 women in the comparison group. There was no difference in socioeconomic status between women with SLE and population controls at the time of the survey (cases were matched on district of residence); nor was there evidence of a difference in level of education attained (*P *= 0.60), although there was some evidence that a greater proportion of SLE cases had grown up in households lacking running water (a proxy measure of hygiene and socioeconomic status in childhood) (*P *= 0.05). A lower proportion of SLE cases reported they had ever been pregnant than women without SLE (80% versus 91%, *P *= 0.002), although this was probably related to the fact that women with SLE were less likely to have ever married or cohabited (*P *= 0.02). There was no difference in oral contraceptive use between the two groups (*P *= 0.5). Among those women who had ever used the oral contraceptive pill, there was no significant difference in length of use between women with SLE (2.4 years; standard deviation, 4.0) and women without SLE (1.8 years; standard deviation, 3.3) (*P *= 0.08).

**Table 1:** Characteristics of female participants at survey, 1998–2002

	Women with SLE (*n *= 122)	Women without SLE (*n *= 203)	*P *value^a^
Age at survey			0.5
15–24 years	4 (3%)	12 (6%)	
25–34 years	21 (17%)	31 (15%)	
35–44 years	39 (32%)	77 (38%)	
45–54 years	32 (26%)	43 (21%)	
≥ 55 years	26 (21%)	40 (20%)	
Mean (standard deviation) age at interview (years)	44.4 (11.6)	43.4 (12.1)	
Education after 18 years	23 (19%)	29 (14%)	0.6
Running water aged 12 years	77 (63%)	149 (73%)	0.05
Ever smoked	10 (8%)	27 (13%)	0.3
Marital status			0.02
Ever married/cohabited	83 (68%)	161 (79%)	
Other	39 (32%)	42 (21%)	
Hysterectomy reported at survey	15 (12%)	22 (11%)	0.7
Ever used contraceptive pill^b^	66 (55%)	100 (51%)	0.5
Ever been pregnant	97 (80%)	184 (91%)	0.002

^a^From chi-squared test for heterogeneity, detecting differences between women with systemic lupus erythematosus (SLE) and those without SLE. ^b^Two women with SLE and seven women without SLE had missing values for this variable.

The reported reproductive histories of the 97 women with SLE and the 184 women without SLE who had ever been pregnant are presented in Table 2. The overall total number of pregnancies reported per woman was very similar in the two groups, and there was no evidence of a difference in mean age at first pregnancy. There was a suggestion that women with SLE were more likely to miscarry, and they appeared to have greater risk of stillbirth. There was also a suggestion that ectopic pregnancy was more common among women with SLE, although numbers were extremely small. These outcomes were explored in more detail in the multivariate, pregnancy-based analyses reported later in the text.

**Table 2:** Reported pregnancy histories at the time of survey among women who had ever been pregnant

	Ever pregnant women with SLE (*n *= 97)	Ever pregnant women without SLE (*n *= 184)	*P *value^a^
Total number of reported pregnancies per woman			0.50
1	13 (13%)	20 (11%)	
2	20 (21%)	36 (20%)	
3	21 (22%)	47 (26%)	
4	19 (20%)	29 (16%)	
5	10 (10%)	23 (13%)	
≥ 6	14 (14%)	29 (15%)	
Mean (standard deviation) number of pregnancies	3.7 (2.0)	3.7 (2.4)	
Mean (standard deviation) age at first pregnancy (years)	22.1 (4.8)	22.5 (4.8)	0.55
Ever had a live birth – total number of live births reported per woman			0.12
0	7 (7%)	2 (1%)	
1	20 (21%)	34 (18%)	
2	22 (23%)	40 (22%)	
3	24 (25%)	51 (28%)	
4	11 (11%)	25 (14%)	
≥ 5	13 (13%)	32 (17%)	
Median (P5–P95)^b ^live births	2.5 (0–5)	3 (1–5)	
Ever miscarried – total number of miscarriages^c ^reported per woman			0.07
0	51 (53%)	125 (68%)	
1	30 (31%)	35 (19%)	
2	11 (11%)	15 (8%)	
≥ 3	5 (5%)	9 (5%)	
Median (P5–P95)^b ^miscarriages^c^	0.5 (0–3)	0 (0–3)	
Ever had a stillbirth – total number of stillbirths reported per woman			0.01
0	86 (89%)	175 (95%)	
1	7 (7%)	9 (5%)	
≥ 2	4 (4%)	0	
Median (P5–P95)^b ^stillbirths	0 (0–1.25)	0 (0–0.75)	
Ever had an ectopic pregnancy			0.17
0	92 (95%)	180 (98%)	
1	5 (5%)	3 (2%)	
≥ 2	0	1 (0.5%)	
Median (P5–P95)^b ^ectopic	0 (0–1)	0 (0–0)	
**Women with a SLE diagnosis only**			
First pregnancy before SLE diagnosis	78 (80%)	-	*-*
First pregnancy after SLE diagnosis	19 (20%)		
Age (years) at first pregnancy			
Mean (standard deviation) age at first pregnancy if before SLE diagnosis	22.1 (4.6)	-	*-*
Mean (standard deviation) age at first pregnancy if after SLE diagnosis	26.6 (7.0)		
Number of reported pregnancies per woman			
Mean (standard deviation) pregnancies whose first conception was before SLE diagnosis	3.7 (2.1)	-	*-*
Mean (standard deviation) pregnancies whose first conception was after SLE diagnosis	3.6 (2.7)		

^a^From chi-squared test for heterogeneity, detecting differences between women with systemic lupus erythematosus (SLE) and those without SLE. ^b^Fifth to 95th centiles – the central 90% of the distribution. ^c ^Includes missed abortions (missed miscarriages) and blighted ova.

The majority (80%) of women diagnosed with SLE had conceived their first pregnancy prior to their SLE diagnosis – although, on average, the total number of pregnancies reported by those whose first conception was prior to SLE diagnosis was the same as that among women whose first conception was after their SLE diagnosis (Table 2). The average age at first conception was significantly higher among women whose first pregnancy was after their SLE diagnosis (*P *< 0.001), as might be expected (Table 2).

### Pregnancy-based analyses

Table 3 presents the characteristics of all reported singleton pregnancies. The overall proportion of pregnancies ending successfully in live birth was much lower among those reported by women with SLE (73%, versus 83% among pregnancies conceived by women without SLE), particularly when the pregnancies were conceived after SLE diagnosis (42%) – although numbers in this latter group were small.

**Table 3:** Characteristics of all reported singleton pregnancies^a^

	Mother ever diagnosed with SLE			Mother never diagnosed with SLE
		
	Pregnancies conceived before mother's SLE diagnosis	Pregnancies conceived after mother's SLE diagnosis	All pregnancies conceived by mothers ever diagnosed with SLE	
Total number of pregnancies	274 (100%)	78 (100%)	352 (100%)	667 (100%)
Mean (standard deviation) maternal age at conception	25.0 (5.5)	30.1 (5.6)	25.6 (5.8)	26.3 (6.1)
Pregnancy type				
Live birth	224/274 (81.8%)	33/78 (42.3%)	257/352 (73.0%)	553/667 (82.9)
Foetal death	47/274 (17.2%)	43/78 (55.1%)	90/352 (25.6%)	109/667 (16.3%)
First-trimester miscarriage (0–12 weeks)^b^	28/274 (10.2%)	34/78 (43.6%)	62/352 (17.6%)	90/667 (13.4%)
Mid-trimester miscarriage (13–23 weeks)^b^	3/259 (1.2%)	4/49 (8.2%)	7/315 (2.2%)	9/605 (1.5%)
Miscarriage missing gestation	3/274 (1.1%)	1/78 (1.3%)	4/352 (1.1%)	1/667 (0.1%)
Stillbirth (≥ 24 weeks)^c^	13/237 (5.5%)	4/37 (10.8%)	17/274 (6.2%)	9/562 (1.6%)
Ectopic pregnancy	3/274 (1.1%)	2/78 (2.6%)	5/352 (1.4%)	5/667 (0.7%)
Reported gestation of live births (completed weeks)				
All pregnancies ending in live birth	224 (100%)	33 (100%)	257 (100%)	553 (100%)
< 24 weeks	5 (2.2%)	3 (9.1%)	8 (3.1%)	20 (3.6%)
24–31 weeks	2 (0.9%)	3 (9.1%)	5 (2.0%)	2 (0.4%)
32–36 weeks	5 (2.2%)	1 (3.0%)	6 (2.3%)	4 (0.7%)
All preterm (< 37 weeks)	12 (5.4%)	7 (21.2%)	19 (7.4%)	26 (4.7%)
37–42 weeks	212 (94.6%)	26 (78.8%)	238 (92.6%)	526 (95.1%)
42–46 weeks	0 (0%)	0 (0%)	0	1 (0.2%)
Reported gestation of foetal deaths (completed weeks)				
All pregnancies ending in foetal death^b^	47 (100%)	43 (100%)	90 (100%)	109 (100%)
< 8 weeks	17 (36.2%)	25 (58.1%)	42 (46.7%)	63 (57.8%)
8–12 weeks	11 (23.4%)	9 (20.9%)	20 (22.2%)	28 (25.7%)
13–15 weeks	2 (4.3%)	0 (0%)	2 (2.2%)	4 (3.7%)
16–23 weeks	1 (2.1%)	4 (9.3%)	5 (5.6%)	5 (4.6%)
24–36 weeks	6 (12.7%)	4 (9.3%)	10 (11.1%)	4 (3.7%)
≥ 37 weeks	7 (14.9%)	0 (0%)	7 (7.7%)	3 (2.8%)
Missing gestation category	3 (6.4%)	1 (2.3%)	4 (4.4%)	2 (1.8%)

^a^Twin pregnancies excluded. ^b^Four pregnancies ending in miscarriage have the gestational age missing (three conceived prior to systemic lupus erythematosus (SLE) diagnosis; one conceived after SLE diagnosis). ^c^One woman without SLE had missing stillbirth gestation.

Overall 1.6% of all reported live births and stillbirths conceived by women without SLE ended in stillbirth (Table 4). This is comparable with the national stillbirth figure of 1.2% of all births [19], suggesting that women without SLE in the present study were representative of the general population – particularly since the stillbirths in the Trinidad population survey were classified as foetal death at ≥ 28 weeks, so the rates of stillbirth defined as foetal death at ≥ 24 weeks as in the study will be higher.

**Table 4:** Foetal death^a ^among pregnancies^b ^reported by Trinidadian women by systemic lupus erythematosus (SLE) status

	Early miscarriage^a,c ^(0–12 weeks)		Late miscarriage^a,d ^(13–23 weeks)		All miscarriage^a,e ^(0–23 weeks)		Stillbirth^f ^(≥ 24 weeks)		All foetal death^g^	
	
	*n *(%)	Adjusted OR^h ^(95% CI)	*n *(%)	Adjusted OR^h ^(95% CI)	*n *(%)	Adjusted OR^h ^(95% CI)	*n *(%)	Adjusted OR^h ^(95% CI)	*n *(%)	Adjusted OR^h ^(95% CI)
Mother not diagnosed with SLE	90 (13.5%)	1.0 (-)	9 (1.8%)	1.0 (-)	100^i ^(15.0%)	1.0 (-)	9 (16.0%)	1.0 (-)	109 (16.3%)	1.0 (-)
Mother diagnosed with SLE	62 (17.6%)	1.4 (0.6–3.1)	7 (2.2%)	1.9 (0.4–9.5)	73^j ^(20.7%)	1.6 (0.8–3.5)	17 (6.2%)	8.5 (2.5–28.8)	90^j ^(25.6%)	2.4 (1.2–4.7)
Pregnancy conceived prior to SLE diagnosis	28 (8.0%)	1.6 (0.7–3.7)	3 (1.0%)	2.2 (0.4–10.9)	34^k ^(9.7%)	1.9 (0.8–4.2)	13 (4.7%)	8.3 (2.3–29.2)	47^k ^(13.3%)	2.6 (1.3–5.4)
Pregnancy conceived after SLE diagnosis	34 (9.7%)	0.4 (0.1–2.6)	4 (1.2%)	-^l^	39^m ^(11.1%)	0.3 (0.0–2.5)	4 (1.4%)	9.8 (1.7–58.0)	43^m ^(12.2%)	1.1 (0.3–3.3)

^a^Includes missed abortions and blighted ova. ^b^All analyses exclude ectopic and twin pregnancies. ^c^Denominator for all analyses having early (first-trimester) miscarriage as the outcome is all reported pregnancies. ^d^Denominator for all analyses having late (mid-trimester) miscarriage as the outcome is all pregnancies surviving 13 weeks gestation or more. ^e^Denominator for all analyses having all miscarriage as the outcome is all pregnancies. ^f^Denominator for all analyses having stillbirth as the outcome is all live births and stillbirths (excluding miscarriages). ^g^Denominator for all analyses having all foetal death as the outcome is all pregnancies. ^h^All odds ratios (ORs) adjusted for district of residence, maternal age, pregnancy order and smoking 95% CI, 95% confidence interval. ^i^One miscarriage has missing gestation; excluded from analyses of early/late miscarriage. ^j^Four miscarriages have missing gestation; excluded from analyses of early/late miscarriage. ^k^Three miscarriages have missing gestation; excluded from analyses of early/late miscarriage. ^l^Model cannot be fitted – all four cases have missing maternal age (strong predictor of miscarriage, which is needed in the model). ^m^One miscarriage has missing gestation.

Table 4 presents multivariate logistic regression analyses of foetal death, adjusted for district of residence, maternal age, pregnancy order and smoking.

Overall foetal death was over twice as likely among pregnancies conceived by women with SLE than women without SLE (OR, 2.4; 95% CI, 1.2–4.7). There was, however, no strong evidence of an effect of SLE on risk of early miscarriage (OR, 1.4; 95% CI, 0.6–3.1, regardless of time of diagnosis). Odds of late miscarriage were almost doubled among pregnancies conceived by women with SLE than those of women without SLE (OR, 1.9; 95% CI, 0.4–9.5), although the numbers of cases were extremely small and this result was not statistically significant. There was strong evidence of an effect of SLE on stillbirth: the odds of stillbirth were over eight times higher among pregnancies conceived by women diagnosed with SLE compared with that among pregnancies conceived by women never diagnosed with SLE (OR, 8.5; 95% CI, 2.5–28.8).

Once adjusted for maternal age, which acted as a strong confounder, there was no strong evidence to suggest a difference in risk of foetal death between pregnancies conceived before and after SLE diagnosis (Table 4), although the numbers of cases among pregnancies conceived after SLE diagnosis were really too small to draw meaningful conclusions.

After adjustment for district of residence, maternal age, pregnancy order and smoking, there was evidence that ectopic pregnancy was over three-and-a-half times as likely among pregnancies conceived by women with SLE as those conceived by women without SLE (OR, 3.6; 95% CI, 0.7–18.7). This effect appeared much stronger among pregnancies conceived after SLE diagnosis (OR, 7.5; 95% CI, 0.9–62.5). These estimates were not statistically significant but the numbers of cases were extremely small, and power was therefore consequently low (Table 5).

**Table 5:** Ectopic pregnancy and live-born preterm delivery among all pregnancies reported by Trinidadian women by systemic lupus erythematosus (SLE) status

	Ectopic pregnancy^a^		Preterm delivery among live-born infants^b^	
	
	*n *(prevalence %)	Adjusted odds ratio (95% confidence interval)	*n *(prevalence %)	Adjusted odds ratio (95% confidence interval)
Mother not diagnosed with SLE	5 (0.7%)	1.0 (-)	26 (4.7%)	1.0 (-)
Mother diagnosed with SLE	5 (1.4%)	3.6 (0.7–18.7)	19 (7.3%)	1.5 (0.7–3.2)
Pregnancy conceived prior to SLE diagnosis	3 (0.8%)	3.1(0.5–17.9)	12 (4.7%)	1.2(0.5–2.8)
Pregnancy conceived after SLE diagnosis	2 (0.6%)	7.5 (0.9–62.5)	7 (2.7%)	3.4 (1.2–10.0)

^a^Denominator for all analyses having ectopic pregnancy as the outcome is all reported pregnancies; adjusted for district of residence, maternal age and smoking. ^b^Denominator for all analyses having preterm delivery as the outcome is all live births (excluding foetal death); adjusted for adjusted for district of residence, maternal age and smoking.

After adjustment for district of residence, maternal age, pregnancy order and smoking, the odds of preterm delivery were also raised – pregnancies conceived by women with SLE being an estimated 50% more likely to be preterm than those conceived by women without SLE (OR, 1.5; 95% CI, 0.7–3.2) (Table 5). This effect again appeared much stronger among pregnancies conceived after SLE diagnosis (OR, 3.4; 95% CI, 1.2–10.0).

Among women who reported three or more pregnancies, there was no apparent difference in risk of three or more miscarriages (any gestation) between women with SLE and without SLE (6/64 (9.4%) versus 9/128 (7.0%), respectively; *P *= 0.6). Nor was there evidence of a difference in risk of three consecutive miscarriages in this group (4/64 (6.3%) among women with SLE versus 7/128 (5.5%) among women without SLE, *P *= 0.8). The numbers, however, were too small to draw firm conclusions.

Our analyses of IgG ACL levels or IgM ACL levels among women with SLE did not show evidence of high IgG ACL or IgM ACL associated with sporadic mid-trimester miscarriage. There was evidence, however, of an association of high titres of IgG ACL and IgM ACL among the small number (*n *= 5, 5%) of women with SLE reporting three or more miscarriages (*P *= 0.001 and *P *= 0.009, respectively, when compared with (normal) expectation), and among women with SLE reporting three consecutive miscarriages (*P *= 0.03 and *P *= 0.05, respectively). Of those women with SLE who experienced a stillbirth, 1/17 (6%) had high IgM ACL and 3/17 (18%) had high IgG ACL. We also found an association of high titres of IgM ACL with preterm delivery (*P *= 0.013) and with ectopic pregnancy (0.05), although this was based on small numbers. Elevated IgG ACL levels were also associated with increased risk of ectopic pregnancy (*P *= 0.03).

Regression analyses adjusting for other socioeconomic indicators such as years of education or previous foetal death made little difference to the overall results reported above. Furthermore, the results were largely unchanged when we used the Sydney antiphospholipid syndrome criterion of loss at > 10 weeks gestation to define the reported foetal losses in this study.

## Discussion

The present study is the first to examine pregnancy outcome in women with SLE in Trinidad, where large family sizes and early age of reproduction make it an ideal environment to study the relationship between SLE and foetal outcomes. Access to healthcare was equal for women with SLE and for women in the comparison group, even in low-income groups.

We found that pregnancies conceived by Afro-Caribbean Trinidadian women with SLE were more likely to end in foetal death (OR, 2.4; 95% CI, 1.2–4.7). The strongest effect was on risk of stillbirth, where pregnancies conceived by women with SLE were greater than eight times more likely to end in this way (OR, 8.5; 95% CI, 2.5–28.8). There was also some evidence of an increased risk of ectopic pregnancy among pregnancies conceived by women with SLE. Finally, we found that pregnancies conceived by women with SLE also appeared more likely to end in preterm delivery. This effect was strongest among those conceived after SLE diagnosis, which were greater than three times more likely to end in preterm delivery than those conceived by women without SLE (OR, 3.4; 95% CI, 1.2–10.0).

Details regarding pregnancy outcomes in general within Trinidad are unavailable, but recent national data suggest that the overall crude live birth rate of 18,490 per 629,315 (2.9%) total female population [19] is approximately twice as high as in North America [27]. At the time of writing there were no national data available on miscarriage in Trinidad, although the national stillbirth rate was estimated as 1.2% total births, which is slightly higher than European figures (approximately 0.5% of total births around the time of this study) [28].

The live birth rate (42%) among women conceiving after a diagnosis of SLE is lower than that reported in other studies (84–87%) [1,3,5]; however, this may reflect higher disease activity and comorbidity in Trinidad. Furthermore, termination of pregnancy is currently illegal in Trinidad and therefore is likely to have been misreported as miscarriage – combined with the fact that most foetal deaths were reported as first-trimester miscarriage (17.6%), this misreporting might suggest that the higher pregnancy loss rate in our study could be explained by a higher proportion of induced abortion in this age group. Our findings for foetal death overall (24% in women diagnosed with SLE and 16% in women without SLE) is slightly higher than those in other studies (7–16%) [1,3,5,8]. Early foetal loss may not, however, have been counted in these studies. Few other studies have reported on ectopic pregnancy in relation to SLE (or it is not clear whether ectopic was included as 'foetal loss') – but an increased risk of ectopic pregnancy was found in SLE patients in one study that did, based on a study of only 17 women (42 pregnancies) with SLE [29]. The increased risk of ectopic pregnancy is possibly explained by reduced tubal motility.

First-trimester miscarriage rates are reported as 18% in women with SLE and 13% in women without SLE, although these may be overestimated values owing to the inclusion of induced abortions, as discussed above. North American studies report first-trimester miscarriage as between 7% and 10% based on moderate numbers of pregnancies in women with SLE (63–267 pregnancies) [1,3,5]. Other European studies have reported similar proportions to those found here. Evidence of first-trimester miscarriage has been reported as 5% in a cohort of 46 women with SLE (60 pregnancies); however, this was in a tertiary centre with intensive management protocol for these high-risk pregnancies [6]. A study in France also estimated a spontaneous miscarriage rate of 16% in European women with SLE in 62 pregnancies (38 women) [12], although this included cases with SLE nephropathy. Spontaneous (first and second) miscarriage rates in pregnant women from Saudi Arabia with lupus nephropathy (inactive and active) are reported as between 25% and 32%, with higher rates in patients with antibodies to phospholipids, although this study included only 24 patients [10].

High stillbirth rates in women with SLE have been reported in a number of other studies, with particular high risk in patients with lupus nephritis and/or altered immunological profiles including antiphospholipid syndrome [5,10,12,16,17]. Some studies include a definition of stillbirth as spontaneous abortion at gestation ≥ 20 weeks [12,17], however, which will inflate the estimates. In a study that defined stillbirth as spontaneous abortion at > 24 weeks (as used here), the prevalence is estimated as 3/19 (16%) pregnancies in women with lupus nephritis. Risk of stillbirth also appears to be increased in women who became pregnant after SLE was diagnosed (3/44 (6.8%) compared with one out of 10 pregnancies before SLE was diagnosed) [7]. In general these figures are difficult to interpret as estimates are based on very low numbers studied, but the general finding of an increased risk of stillbirth is similar to that reported here.

Other studies have identified risk of preterm birth in women with SLE [6-9,12], although in some cases prematurity can be medically induced due to complications such as maternal preeclampsia [14]. We did not analyse cause of preterm birth separately in this study.

Although the present study showed an important relationship between SLE and both foetal death and preterm birth, the study does have limitations, which need to be considered.

First, we were only studying women with SLE who had survived, and it is unknown what the reproductive outcomes of those (more severe cases) who died before survey might have been, and whether this might have impacted onto the results.

Next, there was a differential response rate in cases (93%) and control individuals (70%) in the case – control study from which these data came; however, analyses of socioeconomic status such as car and home ownership did not differ between control individuals who agreed to participate and those that refused. It is possible that there may be bias from residual differences between controls who responded and those that did not; however, after obtaining a household listing of all inhabitants, a strict protocol was followed to identify a possible control. Every attempt was made to ensure that selection included working cases (up to three calls were made to identify eligible control individuals at different times of the day and weekends). No SLE cases or controls were excluded because they were pregnant at the time of the survey.

In epidemiological studies, the capture – recapture technique examines the degree of overlap between two (or more) methods of ascertainment and uses a simple formula to estimate the predicted total size of the population. From capture – recapture methods, we estimate a total of 373 cases of SLE are resident in Northern Trinidad; this includes both sexes. In reality, this is likely to be an underestimate as the probability of being on the dermatology register and on the books of the Lupus Society is associated with one another. Assuming 90% of SLE cases are female, we estimate a total of 335 female adult cases in Northern Trinidad. Allowing for incorrect addresses, exclusions on nonAfro-Caribbean individuals and deaths, the total estimated number of eligible SLE cases in Northern Trinidad is 167, of whom we have ascertained 122 (73%).

Although there is potential over-reporting of 'miscarriage', as termination for nonmedical reasons is illegal, this is unlikely to be differential between women with SLE and women without SLE. We are aware that this misclassification is likely to inflate estimates of foetal loss, particularly first-trimester miscarriage.

Our analyses did not show evidence of either high titres of IgG ACL or IgM ACL with sporadic mid-trimester miscarriage, foetal loss, or stillbirth among SLE cases. There was evidence of an association of high titres of IgG ACL and IgM ACL among women reporting three or more miscarriages, and among women with three consecutive miscarriages, but this was experienced by just five of the 97 women with SLE who reported ever being pregnant. We also found an association of high IgM ACL and IgG ACL with ectopic pregnancy, although this was based on small numbers, and an association of high IgM ACL with preterm delivery. Treatment of women with SLE during pregnancy with aspirin and/or heparin is not, however, standard clinical practice in Trinidad. These findings suggest that raised ACL antibodies cannot explain the results for foetal death reported in the present paper, although the investigation of the role of ACL antibodies in preterm and ectopic pregnancies warrants further attention.

Only 27/122 (22%) women who had ever been pregnant had ever had a history of nephritis; however, specific information at time of pregnancy was not available. No women without SLE had documented renal involvement. In any case, adjusting for history of renal involvement in the analyses made little difference. The percentage of immunosuppressive medication in women with SLE treated with corticosteroids, treated with other immunosuppressive medication, and treated with nonsteroidal antiinflammatory drugs was 96%, 84% and 52%, respectively – compared with values for control individuals of 4%, 16% and 48%, respectively. The prevalence of diagnosed hypertension was higher in control individuals than in cases (56% versus 44%), although this was not significant.

## Conclusion

This well-conducted large population-based case-control study of SLE provided data on 1,029 pregnancies, which allowed us to examine adverse pregnancy outcomes in a systematic way. In women with SLE, we found a significantly increased risk for still birth and all foetal death (not accounted for by raised titres of ACL). We also found evidence for increased (nonsignificant) risk of all miscarriage, and ectopic pregnancies. Risk of preterm birth was significantly increased among pregnancies conceived after SLE diagnosis.

Further studies may help clarify whether ectopic pregnancy risk is raised in women with SLE, and the role of ACL in preterm and ectopic pregnancies. Women with SLE known to have APL syndrome which is associated with recurrent fetal loss and preterm delivery may benefit from antithrombotic treatment including low dose aspirin [31], where early identification of patients of particular risk (e.g. APL syndrome) for closer patient management between rheumatologist and obstetrician may help improve outcomes further. We suggest further work could be carried out in monitoring of high obstetric risk in women with SLE.

## Abbreviations

ACL = anticardiolipin antibody (subtypes IgM and IgG); Anti-ds DNA = Anti-double stranded DNA; APS/APL = Anti-Phospholipid Syndrome; 95% CI = 95% confidence interval; OR = odds ratio; SLE = systemic lupus erythematosus.

## Competing interests

The authors declare that they have no competing interests.

## Authors' contributions

MM and ALP conceived of and designed the study, and acted as supervisors. MM obtained and analysed the data, and prepared the initial drafts. All authors made significant contributions to the manuscript regarding the content and interpretation, and read and approved the final manuscript.
